# Learning from imbalanced fetal outcomes of systemic lupus erythematosus in artificial neural networks

**DOI:** 10.1186/s12911-021-01486-x

**Published:** 2021-04-13

**Authors:** Jing-Hang Ma, Zhen Feng, Jia-Yue Wu, Yu Zhang, Wen Di

**Affiliations:** 1 Department of Obstetrics and Gynecology, Ren Ji Hospital, School of Medicine, Shanghai Jiao Tong University, Shanghai, China; 2 Shanghai Key Laboratory of Gynecologic Oncology, Ren Ji Hospital, School of Medicine, Shanghai Jiao Tong University, Shanghai, China; 3 State Key Laboratory of Oncogenes and Related Genes, Shanghai Cancer Institute, Ren Ji Hospital, School of Medicine, Shanghai Jiao Tong University, Shanghai, China; 4 First Affiliated Hospital of Wenzhou Medical University, Wenzhou, China

**Keywords:** Systemic lupus erythematosus, Imbalanced data, Fetal outcome, Artificial neural networks, Clinical decision assistant

## Abstract

**Objective:**

To explore an effective algorithm based on artificial neural network to pick correctly the minority of pregnant women with SLE suffering fetal loss outcomes from the majority with live birth and train a well behaved model as a clinical decision assistant.

**Methods:**

We integrated the thoughts of comparative and focused study into the artificial neural network and presented an effective algorithm aiming at imbalanced learning in small dataset.

**Results:**

We collected 469 non-trivial pregnant patients with SLE, where 420 had live-birth outcomes and the other 49 patients ended in fetal loss. A well trained imbalanced-learning model had a high sensitivity of 19/21 ($90.8\%$) for the identification of patients with fetal loss outcomes.

**Discussion:**

The misprediction of the two patients was explainable. Algorithm improvements in artificial neural network framework enhanced the identification in imbalanced learning problems and the external validation increased the reliability of algorithm.

**Conclusion:**

The well-trained model was fully qualified to assist healthcare providers to make timely and accurate decisions.

**Supplementary Information:**

The online version contains supplementary material available at 10.1186/s12911-021-01486-x.

## Background

Systemic lupus erythematosus (SLE) is a multi-systemic autoimmune disease, affecting predominantly women of childbearing age [1]. The prevalence of SLE is 111.6 per 100, 000 in African-American, 47.5 per 100, 000 in Caucasian, 42.1 per 100, 000 in Hispanic, 24.9 per 100, 000 in Asian and 46 per 100, 000 in China [2]. With a low prevalence across the world, pregnant cases of SLE patients are extremely rare, which limits the predictive power of data-driven models.

The pregnancy of SLE patients is regarded as high-risk events due to high incidence of obstetric complication (such as pre-eclampsia, eclampsia, thrombocytopenia) and SLE flare, which results in adverse maternal and fetal outcomes [3]. Recently, with the deep insights into rheumatology and advances in techniques of obstetric monitoring, most pregnancies of SLE patients can get favorable outcomes, however, fetal loss, including spontaneous abortion, therapeutic abortion and stillbirth, still exists in a certain proportion (8–40$\%$) [3–5]. Fetal loss outcomes are mostly related to exacerbation of SLE and other obstetric complications [3]. Prediction of fetal loss in advance helps obstetricians choose the appropriate treatment and avoid dispensable fetal loss. A variety of factors affect the pregnant outcomes in different degrees, which makes it complex to assess the state of the disease and predict the pregnant outcomes of SLE patients [6, 7]. The severe shortage of data and the imbalanced class of pregnant outcomes, along with the complexity of assessment, lead to bare investigations on the prediction of fetal loss.

Artificial neural network (ANN) [8–11], a mathematical model in machine learning mimicking the human neural architecture of the brain, describes complex statistical relations between the output and input via densely interconnected simple artificial neurons. The network is usually arranged in a multilayer structure, including input layer, hidden layer and output layer, and is mainly used as a classifier. It is designed to find deep connections within datasets and provides indispensable tools for intelligent medical data analysis [9, 12–14].

With scarce clinical samples and imbalanced pregnant outcomes of pregnant SLE patients, conventional algorithms including ANNs, can not identify the minority of pregnant women with SLE suffering fetal loss outcomes [15]. Such imbalanced learning problem is actually challenging but there is no denying that it makes sense in clinical practice and many other fields [16, 17]. In conventional machine learning algorithms, dealing with imbalanced data is regarded as a 10 challenging problem in data mining research [16]. An algorithm ignores the minority in purpose and always predicts the majority, which can win a high accuracy but it learns nothing. Sampling methods are usually used in imbalanced learning applications to balance categories in the training set, since a balanced data set has a better classification performance [18, 19]. Undersampling method removes a large amount of valuable non-trivial data to keep categories balanced [20]. Oversampling method generates multiple similar copies with the minorities, which may exaggerate noisy information and dilute the important features of the original minorities’ [21, 22]. There are also some modified or updated versions of resampling methods, such as Cluster-Based Over Sampling method [23] and a well-known oversampling approach called Synthetic Minority Over-sampling TEchnique (SMOTE) [24–26].

An alternate approach to imbalanced learning is the tree-based ensemble methodology which integrates several classifier modules to aggregate their predictions. Ensemble methodologies, such as bagging-based, boosting-based, gradient tree boosting algorithms and extreme gradient boosting (XGBoost) have good performances in some specific situations [27–33]. In the neural network framework, several methods perform a heuristic mathematical exploration, such as penalizing the objective function, learning rate adjustment and minimization of misclassification costs [34, 35]. In our work, we integrated the thoughts of comparative and focused study into the neural network to analyze the imbalanced fetal outcomes of pregnant women with SLE and distinguish the minority of positives (fetal loss) from the majority of negatives (live birth).

## Methods

### Patients

A retrospective collection of pregnant SLE patients with an electronic medical records (EHR) was performed in the Department of Obstetrics and Gynecology, Ren Ji Hospital Affiliated to Shanghai Jiao Tong University School of Medicine from September 2011 to June 2018.

- Inclusion Criteria: Pregnant patients with SLE who had established archives and labored in Ren Ji Hospital from September 2011 to June 2018; no limitation to age and gestational weeks; SLE classification diagnostic criteria were according to the 1997 American College of Rheumatology (ACR) revised SLE classification criteria [36].
- Exclusion criteria: Multiple gestation or abortions due to personal reasons were excluded.
  469 pregnant women with SLE who met the above criteria had been included in this study. Among them, 49 cases had fetal loss, and the remaining 420 cases had live births, with a fetal loss rate of $10.4\%$. 338 samples from September 2011 to May 2017 were used for training and internal validation. In consideration of the divisibility and the rough ratio 7 : 3 of training samples to internal validation samples [37], random 234 out of the 338 samples were used for training and the remaining 104 samples were for internal validation. After the model was trained well and wrapped as a clinical decision assist, 131 (11 patients had fetal loss outcomes) latest pregnant SLE patients from June 2017 to June 2018 were collected and ran in the model as external validation. All patients were assessed at least once a month by an experienced obstetrician and by a rheumatologist at least once every trimester.

### Medical definitions

- Live birth: the birth of a living baby [38].
- Fetal loss: defined as all pregnancies that did not end with live birth [5], including spontaneous abortions, therapeutic abortions, stillbirths or intrauterine fetal deaths.
  - Spontaneous abortion: spontaneous termination of a pregnancy before 28 weeks of gestation [39].
  - Therapeutic abortion: abortion for therapeutic reasons as the pregnancy might threat maternal health, such as a life-threatening SLE flare or other severe obstetric complications [40]
  - Stillbirth or intrauterine fetal deaths: any baby born without signs of life after 28 weeks of gestation [41]
- Pre-gestational SLE status—Remission stage: a patient takes a low dose of or has stopped prednisone treatment without clinical manifestations of SLE activity for more than 6 months prior to conception [42]
- Pre-gestational SLE status—Active stage: a patient presents clinical manifestations of SLE activity. Disease activity was evaluated according to the SLE Disease Activity Index 2000 (SLEDAI-2K) [43]
- Pre-gestational SLE status—Initial onset: a new onset or diagnosis of SLE during pregnancy
- SLE clinical manifestations: including nephritis, cutaneous lesion, hematological disorder, arthritis, serositis [36]
  - Nephritis: proteinuria $> 0.5 g/24h$ or Cr.CL. $< 60 ml/min/1.73 m^2$ with active urinary sediment
  - Cutaneous lesion: including malar rash, discoid rash, photosensitivity, oral ulcers
  - Hematological disorder: including hemolytic anemia with elevated reticulocytes, leukopenia $< 4000/mm^3$, lymphopenia $< 1500/mm^3$, thrombocytopenia $< 100,000/mm^3$
  - Arthritis: nonerosive arthritis $\le 2$ peripheral joints, characterized by pain, tenderness or swelling
  - Serositis: pleural effusion, pericarditis

### Data processing

SLE-affected pregnant patients with a live birth outcomes were regarded as negatives and ones with fetal loss outcomes were positives. 29 medical indices from patients’ data (shown in Table 1 ) were selected as inputs, *x*. No data were missing except some in 24-hour-urinary protein level and we filled them in a reasonable way.

Each medical index with continuous real values was normalized to unity and the binary feature was represented as 0 or 1. In such way, the divergence in training was prevented to some degree.

The pre-gestational SLE status was a triple-classified variables, including pre-gestational active stage, remission stage and initial onset during pregnancy. We divided them into 3 independent variables ($x_{11}$, $x_{12}$ and $x_{13}$) for the neural network.

24-hour-urinary protein level: 199 out of 469 pregnant SLE did not have records of 24-hour-urinary protein level test, because for patients whose urinary-protein level in routine urine test is below 30*mg*/*dl*, obstetricians would not prescribe 24-hour-urinary protein level test. If one’s 24-hour-urinary-protein level was blank or below 0.5*g*/24*h*, the value would be set as 0, as the level above 0.5*g*/24*h* means renal damage [44, 45]. Mathematical expression is as follows:

1 $$\begin{aligned} x_{26} = \left\{ \begin{array}{*{20}l} {\textit{Proteinuria}}-0.5 &{}{\textit{Proteinuria}}>0.5 \\ 0 &{}{\textit{Proteinuria}} \le 0.5 \\ \end{array} \right. \end{aligned}$$

### Imbalanced learning model establishment integrating comparative and focused study

**Fig. 1:**
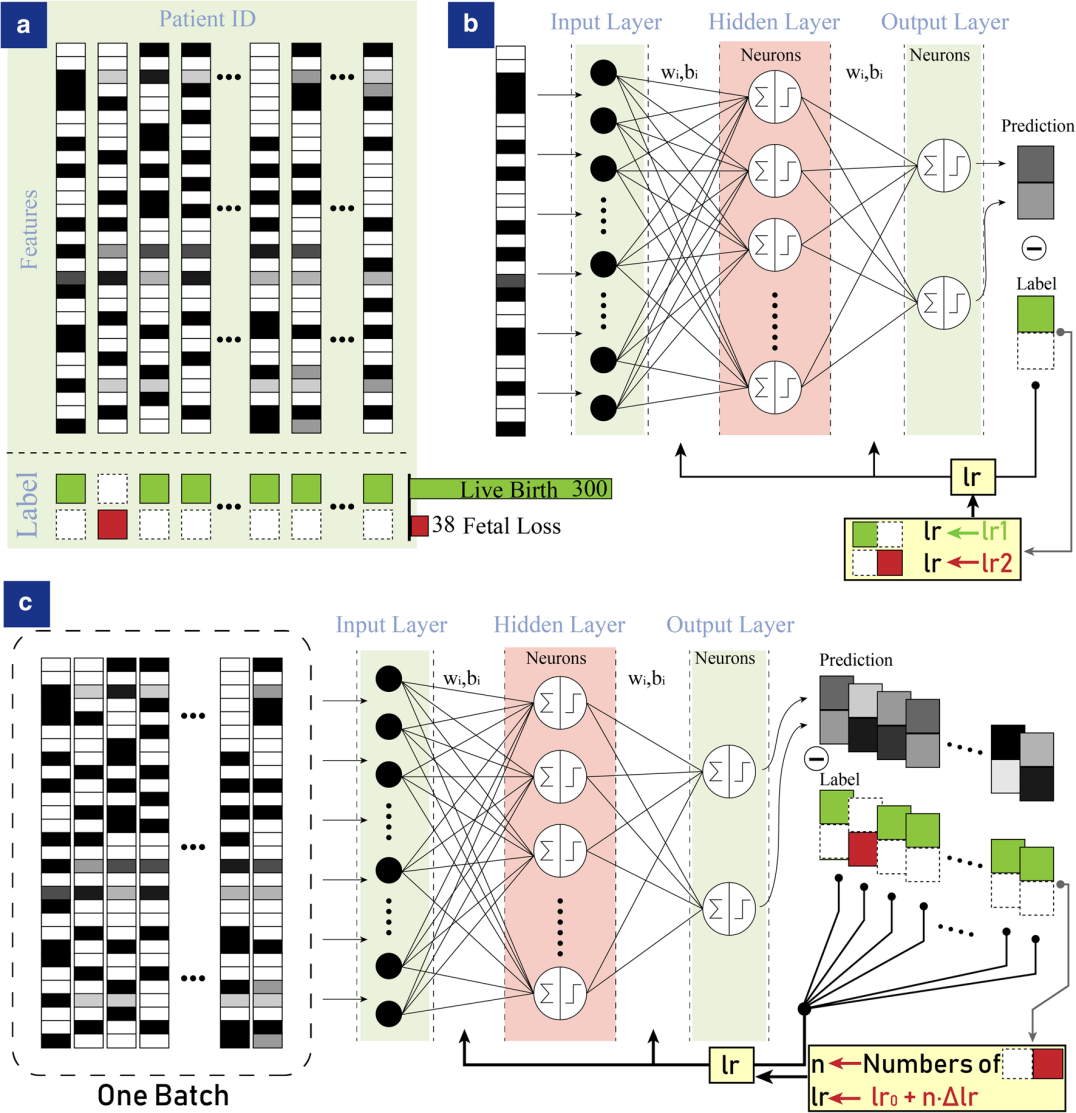
Sample features and learning algorithms. **a** 29 medical indices of one SLE patient were arranged in one column. The label was denoted in green when the SLE patient had a live birth and it was red when the patient had a fetal loss. However, these two classes had largely different quantities where only 38 patients labeled fetal loss in all the 338 patients. **b** Each sample was fed into the artificial neural network (ANN), where the learning rate was dependent on the sample label. **c** Every several samples were trained simultaneously in one batch in the ANN, where the learning rate positively depended on the number of fetal loss samples in the batch

Seminal articles [10, 13, 46, 47] on ANN provided a comprehensive and practical introduction to the conventional neural networks algorithm. In our ANN framework, each patient’s medical records with 29 medical indices (Including baseline characteristics, history, clinical manifestation, laboratory data and treatments) were expressed mathematically by a 29-dimensional input vector $\mathbf {x}$. The corresponding category or class of each patient sample was labeled (positives: fetal loss; negatives: live birth), as illustrated schematically in Fig. 1a.

The learning rate is an important configurable hyperparameter which controls the step size of change in response to the estimated error at each iteration. Conventionally, one sample propagates through the network and produces training error. For an imbalanced-learning problem, the learning rate is binary, where it is *lr*1 when the sample is from the majority, and it increases to *lr*2 when the sample is from the minorities (Fig. 1b). However, training one sample at each iteration tends to predict the majorities and the features of the minorities are hard to be extracted. We first introduced the thoughts of comparative study and combined a number of training samples into a batch to work through before the model’s weights are updated. We then counted the number of minority samples in each batch and introduced the thoughts of focused study. The more fetal loss (minority) samples were in one batch, the more focuses (referring to a higher learning rate) should be concentrated on, since intrinsic distinctions were formed by comparison with samples of different categories.

In our work, we integrated such thoughts of comparative and focused study [8, 34, 35, 48] into the ANN (Fig. 1c) and the network tuned the learning rate dynamically and continuously according to the number of minorities in a batch. For each network, we randomly split the 338 samples into 234 (approximately $70\%$) for training and the other 104 for internal validation. The training set was divided into batches and all samples in one batch were fed into the network together. Learning rate lr was positively related to the number of fetal loss cases in that batch:

2 $$\begin{aligned} lr(i)=lr_0+n(i)\times \Delta lr \end{aligned}$$

*n*(*i*) represented the number of fetal loss samples (labeled positive) in the *i*th batch. In consideration of divisibility, we set the batch size as 13 in this paper.

**Table 1:** 29 medical indices in input neurons

Neurons		Inputs
$x_{01}$	Baseline characteristics	Age
$x_{02}$		Region (city/rural)
$x_{03}$	History	History of live birth* (frequency) (city/rural)
$x_{04}$		History of spontaneous abortion* (frequency)
$x_{05}$		History of therapeutic abortion* (frequency)
$x_{06}$		History of artificial abortion* (frequency)
$x_{07}$		Other adverse reproductive history irrelevant to SLE (frequency)
$x_{08}$		History of caesarean (frequency)
$x_{09}$		Other chronic disease: Diabetes/Hypertension (Y/N)
$x_{10}$		History of SLE (years)
$x_{11}$		Pre-gestational SLE status Remission stage (Y/N)
$x_{12}$		Pre-gestational SLE status Active stage (Y/N)
$x_{13}$		Pre-gestational SLE status Initial onset (Y/N)
$x_{14}$	Clinical manifestation	Nephritis (Y/N)
$x_{15}$		Cutaneous lesion (Y/N)
$x_{16}$		Hematological disorder (Y/N)
$x_{17}$		Arthritis (Y/N)
$x_{18}$		Serositis (Y/N)
$x_{19}$	Laboratory data	Anti-Ro/SSA (Positive/Negative)
$x_{20}$		Anti-La / SSB (Positive/Negative)
$x_{21}$		Anti-dsDNA (Positive/Negative)
$x_{22}$		Anti-Sm (Positive/Negative)
$x_{23}$		APL (Positive/Negative)
$x_{24}$		C3 hypocomplementania-C3 (g/L)
$x_{25}$		C4 hypocomplementania-C4 (g/L)
$x_{26}$		24-hour-urinary protein level (g/L)
$x_{27}$		ADP($\%$)
$x_{28}$	Treatments	Glucocorticoid (Y/N)
$x_{29}$		Asprin (Y/N)

*Medical terms are shown in the Method section

### Evaluation indices

Aiming at finding fetal loss patients to the greatest extent, we set sensitivity, which measures the percentage of pregnant patients with fetal loss who are correctly identified, as the most significant evaluation index. Accuracy is also an important index computing the correct prediction percentage all over samples. Specificity is the extent to which actual negatives are correctly identified as such. Some other metrics like F1-score, Precision Recall Area Under Curve (PR-AUC) and Matthews correlation coefficient (MCC) are also used as the evaluation indices.

Stochastic optimization algorithm, concretely speaking stochastic gradient descent, is used to train the neural network with randomly initialized weights. In the phase of parameter establishment, to overcome the contingency and generalize the results, we trained about 120 neural networks with both random initialization of weights and randomly-selected samples. For each network, random 234 out of 338 samples were put in the training set and the remaining 104 ones, not used for training, were tested and predicted in the calculation of evaluation indices (Sensitivity, specificity or accuracy). Some networks, which were not convergent in the training process or performed totally wrong test results, were excluded and then we averaged the remaining indices to determine network parameters.

Given optimal parameters (learning rate, hidden neurons, etc.), we trained thousands of neural networks in the phase of classified prediction and picked the one with a high sensitivity and comprehensive consideration of accuracy and specificity.

### Hidden neuron configuration

In ANN, each neuron in the hidden layer conducts a nonlinear function on the input and learns some knowledge by mapping from medical indices to predictive categories. Redundant neurons lead to an over-fitting while insufficient ones give an incomplete expression. In order to establish the number of artificial neurons in the hidden layer, we selected sensitivity as the key indicator. Given $\Delta lr$, we trained 120 neural networks for each configuration and averaged their sensitivities as the performance parameter, where the optimum determined the number of neurons in the hidden layer.

### Decision threshold

In the previous literature, cost-sensitive learning was used to modify the cost of misclassification in the decision process [34]. We made a novel adjustment applied easily to the ANN framework to have almost the same effect. Extracting values from the two corresponding output neurons, we obtained two outputs $o_1$ and $o_2$, and they met the normalization constraint:

3 $$\begin{aligned} o_1+o_2=1 \end{aligned}$$

In a general condition, the positive (minority) category is predicted if $o_1>0.5>o_2$, and vice versa. In fact, it is very hard to predict positives with enough confidence due to the shortage of its samples. The classification criteria could be changed in dependence on the quantity contrast of two categories. We set $\Delta$ as the variation of decision threshold, which meant the minorities or positives were predicted if $o_1>0.5-\Delta$ (Fig. 4b).

## Results

### Evaluation by a conventional ANN method in imbalanced learning

The seminal work of dealing with imbalanced data made cost-sensitive modifications of the back-propagation learning algorithm in the ANN framework [34] and the schematic graph was shown in Fig. 1b. Each sample was fed into the ANN and the network decides the learning rate *lr* based on the label of this sample:

4 $$\begin{aligned} {\textit{lr}} = \left\{ \begin{array}{*{20}l} { lr_0 } &{} { label_i=negative } \\ lr_0 + \delta lr &{} { label_i=positive } \\ \end{array} \right. \end{aligned}$$

**Fig. 2:**
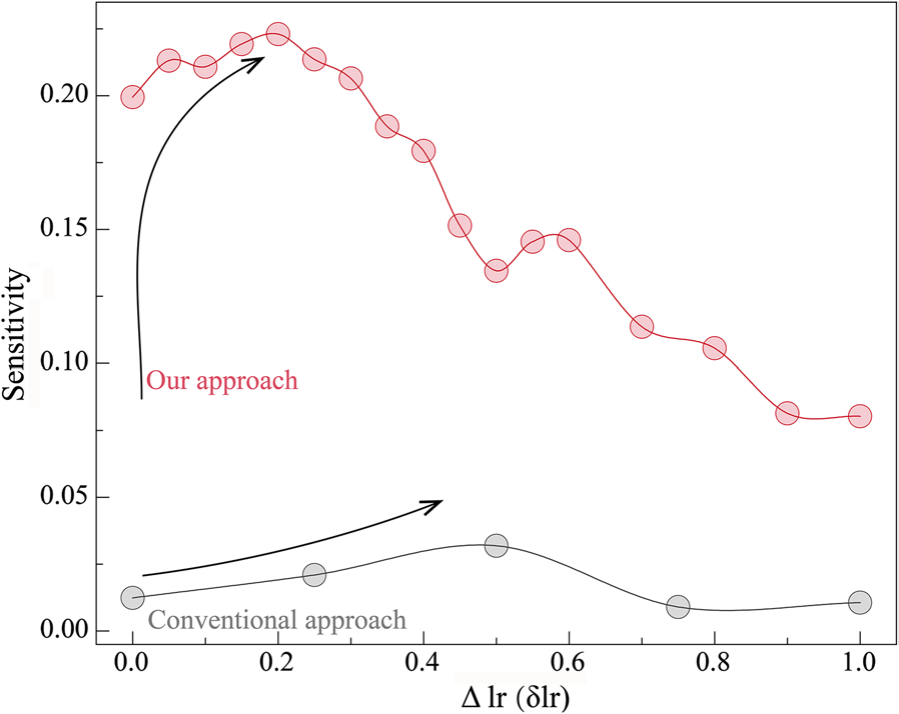
The relation between $\Delta lr$ and sensitivity. Red curve referred to our algorithm shown in Fig. 1c, $\Delta lr$ reaches the optimum when it was around 0.2. Gray curve showed the results according to the algorithm in Fig. 1b

$lr_0$ was assigned as 1. We performed a proof-of-principle demonstrations by assigning $\delta lr$ from 0 to 1 spacing 0.2. We calculated the sensitivity of each $\delta lr$ and showed them in the gray curve in Fig. 2. Presetting 8 artificial neurons in the hidden layer in the 3-layer ANN empirically, the result indicated that optimal sensitivity was $3.5\%$ with scanning $\delta lr$ and equivalently fewer than one out of twenty positive samples could be picked correctly on average.

### Integrating the thoughts of comparative and focused study into the neural network framework

Figure 2 showed the relation between $\Delta lr$ and sensitivity. Integrating the thoughts of comparative study and focused study, we observed a significant increase from $3.5\%$ to $19.9\%$ and then to $22.3\%$ when $\Delta lr$ was around 0.2.

**Fig. 3:**
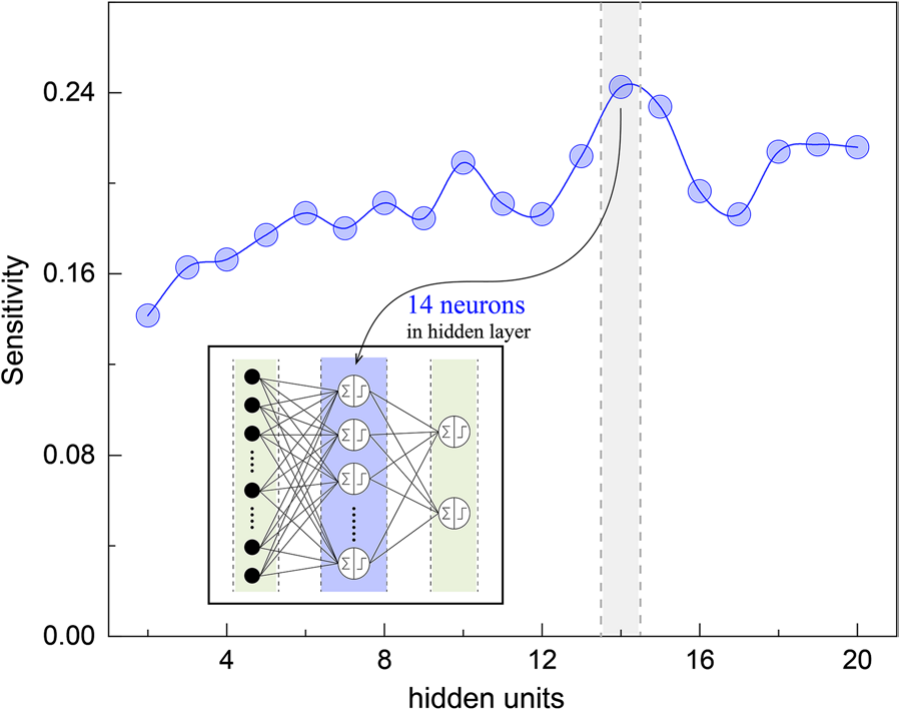
Number of configured artificial neurons in the hidden layer. An optimal number of hidden neurons was selected as 14 which obtained a highest sensitivity. Inset: ANN configuration

Subsequently, we established the number of artificial neurons in the hidden layer in Fig. 3 and the inset was the structure of our ANN. A steady rise was found until 14 neurons and then a drop appeared after that, which explained a 14-neuron hidden layer exactly described the mathematical expression bridging inputs and outputs. Therefore, the configuration of 3-layer ANN was assigned as 29-14-2 in substitution of the previous 29-8-2.

**Fig. 4:**
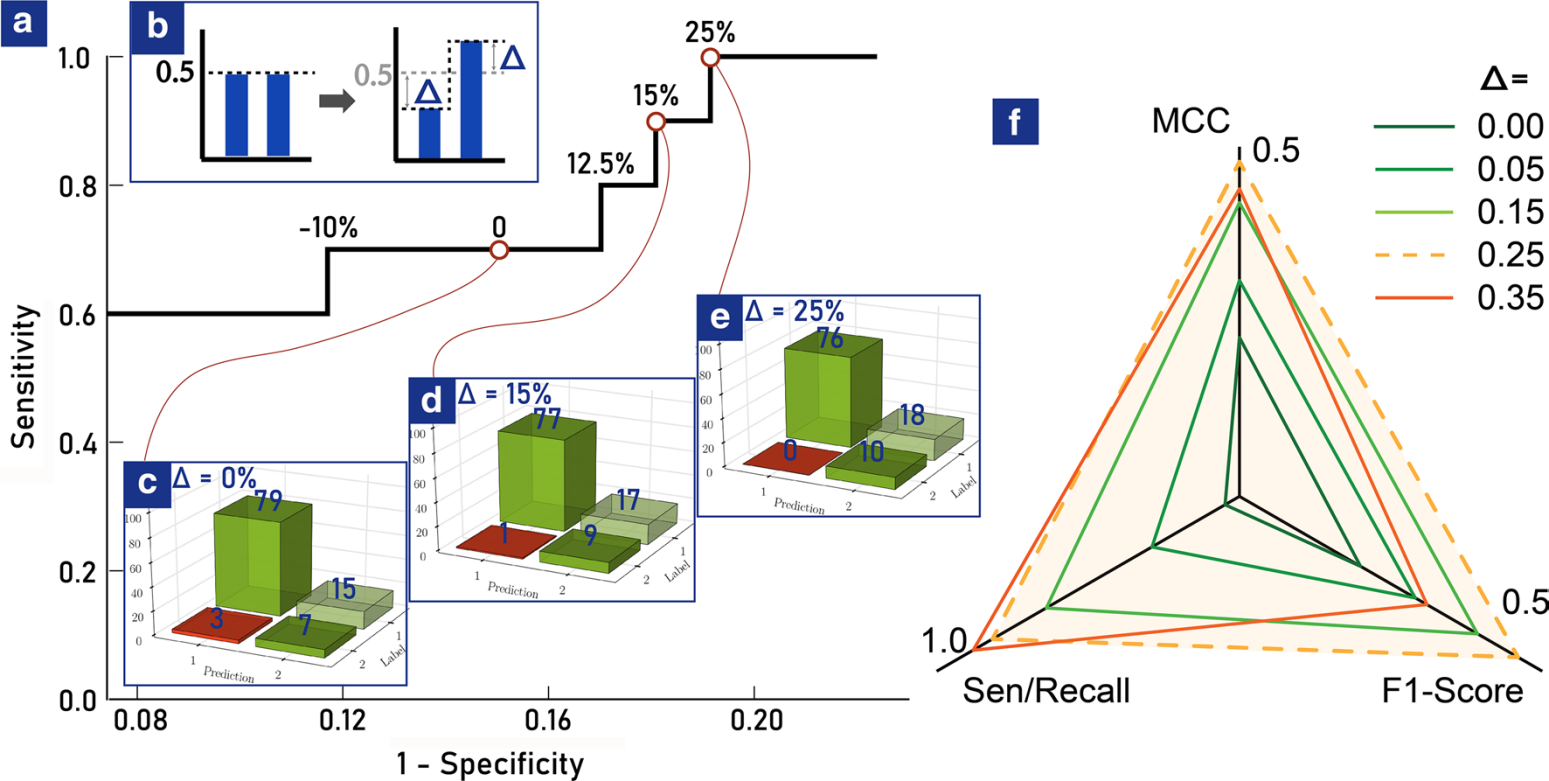
Internal validation of the well-trained ANN models and the strategy of shifting diagnosis threshold. **a** The relation between specificity and sensitivity with varying $\Delta$ from $-20$ to $25\%$. **b** $\Delta$ was the variation of decision threshold and the minorities or positives were predicted if $o_1 > 0.5-\Delta$. **c–e** showed the confusion matrix when $\Delta$ was 0, $15\%$ and $25\%$. Category 1 showed SLE patients with fetal-loss outcomes and Category 2 were ones with live-birth outcomes. **f** visualization of the three metrics in different $\Delta$.

Given the optimal network parameters, we trained thousands of neural networks (without cross validation). Training is stopped to avoid overfitting when the sum squared error on the validation set has begun to rise. We then picked one with high performance (Sensitivity: $70\%$), whose confusion matrix with the internal validation set was shown in Fig. 4c. We found that 7 out of 10 patients with fetal loss outcomes were correctly identified but the other 3 were misdiagnosed.

### Shifting the decision threshold

To improve identification of patients with fetal loss outcomes, we increased $\Delta$ gradually to lower the decision threshold for fetal loss prediction. Figure 4a showed a trade-off between sensitivity and specificity with dynamically varying $\Delta$ from $-20$ to $25\%$ (Dynamic evolution is shown in the Additional file 1: Video 1). Figure 4d and e visualized confusion matrix when $\Delta$ was $15\%$ and $25\%$, respectively. Remarkably, the sensitivity arrived at $100\%$ and the specificity was also over $80\%$ when $\Delta$ was $25\%$. To avoid sample selection bias, we generated optimal models with different input/output combinations and averaged the evaluation indices according to $\Delta$ in Table 2. Among them we visualized the three metrics (i.e. sensitivity, MCC, F1-score) suitable for imbalanced datasets in Fig. 4f. Overall, the well-trained ANN with shifting the threshold by $25\%$ was well qualified and equipped as a clinical decision assistant [49, 50].

**Table 2:** The average evaluation indices for different $\Delta$

$\Delta$	MCC	F1-Score	Sensitivity	Accuracy
0	$0.42 \pm 0.10$	$0.47 \pm 0.09$	$0.67 \pm 0.08$	$0.85 \pm 0.04$
0.05	$0.44 \pm 0.09$	$0.48 \pm 0.08$	$0.75 \pm 0.04$	$0.84 \pm 0.05$
0.15	$0.48 \pm 0.07$	$0.49 \pm 0.08$	$0.87 \pm 0.05$	$0.82 \pm 0.07$
$\mathbf{0} .\mathbf{25}$	$0.49 \pm 0.11$	$0.50 \pm 0.11$	$0.94 \pm 0.05$	$0.81 \pm 0.08$
0.35	$0.48 \pm 0.13$	$0.48 \pm 0.12$	$0.96 \pm 0.05$	$0.79 \pm 0.09$

### External validation

**Fig. 5:**
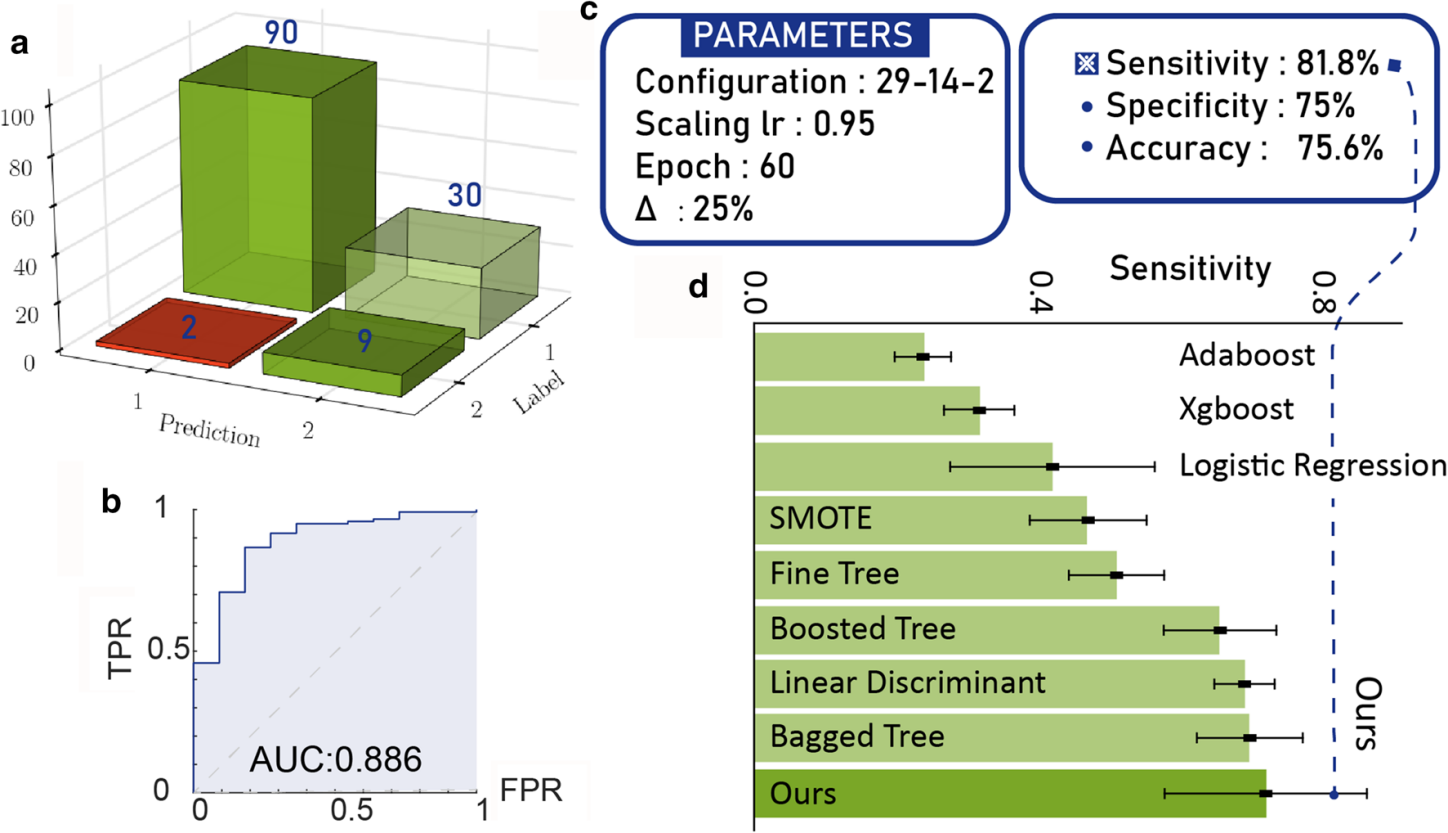
Wrapped clinical decision assistant and the experimental study with newly-obtained samples. **a** 131 newly generated patient data ran into the assistant and the confusion matrix was displayed. Category 1 showed SLE patients with fetal-loss outcomes and Category 2 were ones with live-birth outcomes. **b** the ROC curve and the AUC. **c** showed part of configuration parameters in the wrapped machine. **d** presented the statistical performances of the external validation

To further validate the model, we externally validated the developed network using the latest 131 samples of pregnant patients with SLE from June 2017 to June 2018 treated in the same hospital, other than the 338 ones before May 2017. Figure 5a showed confusion matrix given by our model, where 9 out of 11 SLE patients with fetal loss outcomes were picked correctly from all 131 samples. In fact, the two misdiagnosed fetal-loss patients were explainable and we expanded them in detail in the Discussion section. Besides, the receiver operating characteristic (ROC) curve, depicting the trade-off between true positive rate (TPR) and false positive rate (FPR), was shown in Fig. 5b and the area under the curve (AUC) was 0.886. Statistical performances of the external validation were measured in Fig. 5c.

### Comparison of other models

XGBoost and AdaBoost were firstly implemented in R using xgboost and JOUSBoost package. (XGBoost: maximum depth of a tree: 2,4,6; L2 regularization term:1,2; the learning rate:1; max number of boosting iterations: 500; learning objective: logistic regression for binary classification. AdaBoost: maximum depth of a tree: 1-6, max number of boosting iterations: 500.) We then performed the SMOTE algorithm with nine combinations of the parameters (*percentover*: 500, 600, 700; *k*:3,4,5) using DMwR package in R [24]. We also tried another five classification algorithms applicable to the imbalanced learning problem, such as fine tree, boosted tree, bagged tree, linear discriminant and logistic regression. Accordingly, we choose the sensitivity as the key indicator and error bars are calculated by the standard variance of tens of individual results within each algorithm in Fig. 5d. All of them were below the sensitivity of $81.8 \%$ in our network.

## Discussion

ANN has the “black box” nature, where some parameters and output results cannot be explained intuitively and directly. As SLE is a complex disease and many factors may lead to the fetal loss outcome in pregnant women with SLE [51]. ANN, representing complex statistical relations with densely interconnected neurons, may describe the complex associations of the fetal loss outcome and clinical information.

We further analyzed the 2 patients who were misdiagnosis: *No*.92 Patient had a 10-year SLE history and the assessment before pregnancy indicated the remission stage of illness. Unfortunately, she suffered fetal loss due to antepartum bleeding of placenta previa, tocolytic agents failed to stop colporrhagia, so a caesarean had to be performed to terminate the pregnancy at $22+3w$. This spontaneous abortion was attributed to antepartum bleeding of placenta previa, which had not been reported having relationship with SLE.

The other misdiagnosed patient (*NO*.117) had a 13-year SLE history and her assessment before pregnancy showed a remission stage of illness. During the gestation, her state of illness kept quiescent in clinical manifestation and laboratory test, but she suffered preterm premature rupture of membranes (PPROM) at $20+2w$ and failed to keep the fetus. The previous studies have reported that pregnant SLE patients were liable to get genital infection with long-term use of glucocorticoids and had a higher risk of PPROM than general population [40]. Such case of spontaneous abortion caused by PPROM is very rare and our machine has not learnt similar samples in training or internal validation dataset. Despite this, it could be picked correctly when the decision threshold was shifted onto $35\%$, which illustrated that the algorithm had indeed found deep connections behind input data.

The fetal loss rate in this work was $10.4\%$ (49/469). The low fetal loss rate produced the imbalanced data and brought difficulties in training the machine learning algorithm [15]. The conventional approach tended to be overwhelmed by the majority and ignored the minority to pursue an accurate performance. The thought of comparative study is to divide samples into batches. The randomness of batch arrangement brings robust sample features into the training process. Concretely, randomly matched samples in a batch find a maximum discrimination and determines a resultant descending gradient. Focused study places emphasis on the minorities and customizes the learning rate. This approach improves the flexibility of samples and avoids over-fitting from sample duplication, because the non-discrete learning rate weakens the individual characteristics. Our approach in this study works out the puzzle of dealing with imbalanced data which can be used for reference in dealing with other imbalanced non-trivial medical data.

The application of threshold shift is in accordance with the function and the actual requirement of the algorithm. In this work, to find fetal loss patients as many as possible, we lowered the threshold for fetal loss prediction to distinguish more potential fetal loss patients. The sensitivity, rather than the accuracy, was supposed to be the most important evaluation index, since a higher sensitivity meant less fetal loss patients were in truth omitted.

The external validation was composed by 131 independent patients who had not been used in neither the training process nor the internal validation. The external validation verified the reliability of the predictive model, since constant samples could avoid random selection in training or internal validation set, which leads to accidental high accuracies.

Our work realized a neural-network-based predictor, where the algorithm, compared to conventional neural network, could be applied to predict imbalanced pregnant outcomes and discover potential fetal loss patients. In clinical practice, the number of pregnant patients with SLE that a physician meets is limited, thus it is a difficulty for physicians to predict the fetal loss of patients. This model learnt the experience of hundreds of patients, which is more experienced than physicians to help find the high-risk patients of fetal loss. For clinical application, the prediction can help obstetricians find high-risk pregnant SLE patients who are liable to fetal loss and more severe in the state of illness, as fetal loss is often related to SLE flare. If the algorithm predicts ’fetal loss’ for patients whose fetus have a great probability to survival, intensive monitoring should be taken and the termination of pregnancy in time should be thought to avoid dispensable fetal loss during expectation treatment. For patients who have adjusted treatment or revaluated during pregnancy, the algorithm can re-predict their pregnant outcomes to assess the curative effect or the progression of illness. For patients in early gestation period, if the algorithm predicts ’fetal loss’ with exacerbation of SLE, therapeutic abortion should be considered to prevent life-threaten events.

Our study had some limitations. In Shanghai China, the antenatal care of pregnant women before 12 weeks of gestation was taken at community hospital, the spontaneous abortion of SLE patients are treated at out-patient service. The clinical data of these patients could not be found in electronic health record (EHR) of the hospital and were not included in this study. Moreover, the black-box nature of the algorithm [52] makes it difficult to interpret the risk factors and their weight in fetal loss of pregnant SLE patients.

## Supplementary Information

**Additional file 1: Video 1.** Trade-off between sensitivity and specificity with dynamically varying Δ from -20% to 25%

## Data Availability

The datasets generated and/or analysed during the current study are not publicly available due to privacy but are available from the corresponding author on reasonable request.

## References

[1] Chakravarty EF, Colón I, Langen ES, Nix DA, El-Sayed YY, Genovese MC, Druzin ML (2005). Factors that predict prematurity and preeclampsia in pregnancies that are complicated by systemic lupus erythematosus. Am J Obstet Gynecol.

[2] Somers EC, Marder W, Cagnoli P, Lewis EE, DeGuire P, Gordon C, Helmick CG, Wang L, Wing JJ, Dhar JP, Leisen J, Shaltis D, McCune WJ (2013). Population-based incidence and prevalence of systemic lupus erythematosus: The michigan lupus epidemiology and surveillance program. Arthr Rheumatol.

[3] Chen S, Sun X, Wu B, Lian X (2015). Pregnancy in women with systemic lupus erythematosus: a retrospective study of 83 pregnancies at a single centre. Int J Environ Res Public Health.

[4] Doria A, Iaccarino L, Ghirardello A, Zampieri S, Arienti S, Sarzi-Puttini P, Atzeni F, Piccoli A, Todesco S (2006). Long-term prognosis and causes of death in systemic lupus erythematosus. Am J Med.

[5] CLOWSE MEGANEB, MAGDER LAURENCES, PETRI MICHELLE (2011). The clinical utility of measuring complement and Anti-dsDNA antibodies during pregnancy in patients with systemic lupus erythematosus. J Rheumatol.

[6] Khamashta MA (2006). Systemic lupus erythematosus and pregnancy. Best Pract Res Clin Rheumatol.

[7] Mok CC, Wong RWS (2001). Pregnancy in systemic lupus erythematosus. Postgrad Med J.

[8] Haykin S (1998). Neural networks: a comprehensive foundation.

[9] Sheikhtaheri A, Sadoughi F, Hashemi Dehaghi Z (2014). Developing and using expert systems and neural networks in medicine: a review on benefits and challenges. J Med Syst.

[10] LeCun Y, Bengio Y, Hinton G (2015). deep learning. Nature.

[11] Ceccarelli F, Sciandrone M, Perricone C, Galvan G, Morelli F, Vicente LN, Leccese I, Massaro L, Cipriano E, Spinelli FR, Alessandri C, Valesini G, Conti F (2017). Prediction of chronic damage in systemic lupus erythematosus by using machine-learning models. PLoS ONE.

[12] Kononenko I (2001). Machine learning for medical diagnosis: history, state of the art and perspective. Artif Intell Med.

[13] Amato F, López A, Peña-Méndez EM, Vaňhara P, Hampl A, Havel J (2013). Artificial neural networks in medical diagnosis. J Appl Biomed.

[14] Shahin M, Ahmed B, Hamida ST, Mulaffer FL, Glos M, Penzel T (2017). Deep learning and insomnia: assisting clinicians with their diagnosis. IEEE J Biomed Health Inform.

[15] Chawla NV, Japkowicz N, Kotcz A (2004). Editorial: special issue on learning from imbalanced data sets. SIGKDD Explor Newsl.

[16] Yang Q, Wu X (2006). 10 challenging problems in data mining research. Int J Info Tech Dec Mak.

[17] Zou J, Huss M, Abid A, Mohammadi P, Torkamani A, Telenti A (2019). A primer on deep learning in genomics. Nat Genet.

[18] Weiss GM, Provost F. The effect of class distribution on classifier learning: An empirical study. Technical report 2001.

[19] Laurikkala J. Improving identification of difficult small classes by balancing class distribution. In: Proceedings of the 8th conference on AI in medicine in Europe: artificial intelligence medicine. AIME ’01. Berlin, Heidelberg: Springer; 2001. p. 63–6.

[20] Liu X, Wu J, Zhou Z (2009). Exploratory Undersampling for Class-Imbalance Learning. IEEE Trans Syst Man Cybern Part B (Cybern).

[21] Batista GEAPA, Prati RC, Monard MC (2004). A study of the behavior of several methods for balancing machine learning training data. SIGKDD Explor Newsl.

[22] Han H, Wang W-Y, Mao B-H, Huang D-S, Zhang X-P, Huang G-B (2005). Borderline-SMOTE: A New Over-Sampling Method in Imbalanced Data Sets Learning. Advances in Intelligent Computing.

[23] Santos MS, Abreu PH, García-Laencina PJ, Simão A, Carvalho A (2015). A new cluster-based oversampling method for improving survival prediction of hepatocellular carcinoma patients. J Biomed Inform.

[24] Chawla NV, Bowyer KW, Hall LO, Kegelmeyer WP (2002). Smote: synthetic minority over-sampling technique. J Artif Intell Res.

[25] Hanifah FS, Wijayanto H, Kurnia A. Smote bagging algorithm for imbalanced dataset in logistic regression analysis (case: Credit of bank x). 2015.

[26] Yan S, Qian W, Guan Y, Zheng B (2019). Improving lung cancer prognosis assessment by incorporating synthetic minority oversampling technique and score fusion method. Med Phys.

[27] Pavlov DY, Gorodilov A, Brunk CA. Bagboo: a scalable hybrid bagging-the-boosting model. In: CIKM. 2010.

[28] Sariyar M, Borg A, Pommerening K (2012). Active learning strategies for the deduplication of electronic patient data using classification trees. J Biomed Inform.

[29] de Matos Simoes R, Emmert-Streib F (2012). Bagging statistical network inference from large-scale gene expression data. PLoS ONE.

[30] Galar M, Fernandez A, Barrenechea E, Bustince H, Herrera F (2012). A review on ensembles for the class imbalance problem: Bagging-, boosting-, and hybrid-based approaches. IEEE Trans Syst Man Cybern Part C (Appl Rev).

[31] Chen T, Guestrin C. Xgboost: A scalable tree boosting system. In: Proceedings of the 22nd ACM SIGKDD international conference on knowledge discovery and data mining. KDD ’16. New York, NY, USA: Association for Computing Machinery; 2016. p. 785–94. 10.1145/2939672.2939785.

[32] Deist TM, Dankers FJWM, Valdes G, Wijsman R, Hsu I-C, Oberije C, Lustberg T, van Soest J, Hoebers F, Jochems A, El Naqa I, Wee L, Morin O, Raleigh DR, Bots W, Kaanders JH, Belderbos J, Kwint M, Solberg T, Monshouwer R, Bussink J, Dekker A, Lambin P (2019). Machine learning algorithms for outcome prediction in (chemo)radiotherapy: an empirical comparison of classifiers. Med Phys.

[33] Govindarajan P, Soundarapandian RK, Gandomi AH, Patan R, Jayaraman P, Manikandan R. Classification of stroke disease using machine learning algorithms. Neural Computing and Applications. 2019.

[34] Kukar MZ, Kononenko I. Cost-Sensitive Learning with Neural Networks. 1998. p. 445–449.

[35] Zhou Z, Liu X. Training Cost-Sensitive Neural Networks with Methods Addressing the Class Imbalance Problem. IEEE Trans Knowl Data Eng. 2006.

[36] Yu C, Gershwin ME, Chang C (2014). Diagnostic criteria for systemic lupus erythematosus: a critical review. J Autoimmun.

[37] Paydar K, Niakan Kalhori SR, Akbarian M, Sheikhtaheri A (2017). A clinical decision support system for prediction of pregnancy outcome in pregnant women with systemic lupus erythematosus. Int J Med Inf.

[38] Velez AMA, Howard MS. Lupus erythematosus: A comprehensive review. In: Lupus: Symptoms, Treatment and Potential Complications. 2012. p. 13–53.

[39] Xie Xing, Gou Wenli, Di Wen, Lin Zhongqiu, KB Ding Ma (2013). Obstetrics and gynecology people’s medical publishing house.

[40] Wu J, Ma J, Bao C, Di W, Zhang WH (2018). Pregnancy outcomes among Chinese women with and without systemic lupus erythematosus a retrospective cohort study. Bmj Open.

[41] Shah NH, Tenenbaum JD (2012). The coming age of data-driven medicine: translational bioinformatics’ next frontier. J Am Med Inform Assoc.

[42] Lateef A, Petri M (2013). Managing lupus patients during pregnancy. Best Pract Res Clin Rheumatol.

[43] Gladman DD, Ibañez D, Urowitz MB (2002). Systemic lupus erythematosus disease activity index 2000. J Rheumatol.

[44] Lv J, Wang W, Li Y (2015). Clinical outcomes and predictors of fetal and maternal consequences of pregnancy in lupus nephritis patients. Int Urol Nephrol.

[45] Moroni G, Doria A, Giglio E, Imbasciati E, Tani C, Zen M, Strigini F, Zaina B, Tincani A, Gatto M (2016). Maternal outcome in pregnant women with lupus nephritis: a prospective multicenter study. J Autoimmun.

[46] Baxt WG (1995). Application of artificial neural networks to clinical medicine. The Lancet.

[47] Jain AK, Mao J, Mohiuddin KM (1996). Artificial neural networks: a tutorial. Computer.

[48] Yin P, Luo P, Nakamura T. Small Batch or Large Batch?: Gaussian Walk with Rebound Can Teach. In: Proceedings of the 23rd ACM SIGKDD international conference on knowledge discovery and data mining. KDD ’17. New York, NY, USA: ACM; 2017. p. 1275–1284. 10.1145/3097983.3098147.

[49] Berner ES, Hannah KJ, Ball MJ (2017). Clinical decision support systems: theory and practice. MCN Am J Maternal/child Nurs.

[50] Huang M, Han H, Wang H, Li L, Zhang Y, Bhatti UA (2018). A clinical decision support framework for heterogeneous data sources. IEEE J Biomed Health Inf.

[51] Smyth A, Garovic V (2009). Systemic lupus erythematosus and pregnancy. Minerva urologica e nefrologica= The Italian Journal of Urology and Nephrology.

[52] Lundberg SM, Nair B, Vavilala MS, Horibe M, Eisses MJ, Adams T, Liston DE, Low DK-W, Newman S-F, Kim J, Lee S-I (2018). Explainable machine-learning predictions for the prevention of hypoxaemia during surgery. Nat Biomed Eng.

